# Validation of Two Kinetic Assays for the Quantification of Endotoxin in Human Serum

**DOI:** 10.3389/fneur.2021.691683

**Published:** 2021-06-25

**Authors:** Christian Barro, Anu Paul, Fermisk Saleh, Tanuja Chitnis, Howard L. Weiner

**Affiliations:** ^1^Ann Romney Center for Neurologic Diseases, Brigham and Women's Hospital, Boston, MA, United States; ^2^Department of Neurology, Harvard Medical School, Boston, MA, United States; ^3^Department of Neurology, Partners Multiple Sclerosis Center, Brigham and Women's Hospital, Boston, MA, United States

**Keywords:** endotoxin, multiple sclerosis, microbiota, assay validation, gut brain axis, neuroinflammation

## Abstract

**Background:** There is an emerging evidence of the role of the microbiome in neurological diseases. Endotoxin is a component of gram-negative bacteria and thought to be one of the possible signals between the gut microbiota and the immune system. Previous studies explored the blood levels of endotoxin using an endpoint chromogenic assay.

**Methods:** We validated and compared the analytical performance of two kinetic assays for the quantification of endotoxin in serum: (1) the Limulus Amebocyte Lysate (LAL) Kinetic-QCL assay and (2) the turbidimetric LAL Pyrogent-5000 assay. We used the best-performing validated assay to measure the endotoxin level in 20 patients with multiple sclerosis (MS) and eight healthy controls.

**Results:** The Pyrogent-5000 and QCL assay achieved similar performance in regard to spike recovery and linear dilution; however, the Pyrogent-5000 had a better signal to noise in the calibrator curve. By using the Pyrogent-5000 assay, we found that serum samples from MS patients and healthy controls have a similar level of endotoxin; hence, we did not find evidence to support a penetration of endotoxin in the blood of MS patients. Our findings do not exclude a role of endotoxin in mediating signals from the gut microbiota in MS patients directly at the gut–blood barrier where numerous antigen-presenting cells are actively sensing metabolites and bacterial products.

## Introduction

The microbiome is increasingly acknowledged for its potential role in conditions such as autoimmune diseases (1). This applies for example to multiple sclerosis (MS) and other neurodegenerative diseases as amyotrophic lateral sclerosis (ALS) and Alzheimer's disease (AD). We have recently shown that MS patients had a different microbiome than healthy individuals and the microbiome differed also between the relapsing–remitting (RRMS) and secondary progressive MS (SPMS) patients (2). Strikingly, the oral gavage of bacteria that were overrepresented in SPMS patients, such as *Akkermansia muciniphila*, caused an amelioration in the disease course of experimental autoimmune encephalomyelitis (EAE)—mouse model used for resembling CNS inflammation in MS (2). In the ALS field, it was recently shown that depleting the gut microbiota in a c9orf72 ALS mouse model caused an improvement in the disease course (3). Despite the growing evidence supporting a role for the gut–brain axis in neurological diseases, there is a lack of understanding on how the communication between the gut microbiota and immune system is mediated. This prompted scientists to explore the pathways by which the microbiome may communicate with our immune system. The most prominent hypothesis is that this communication occurs at the level of the gut–blood barrier between the gut microbiota at the immune system (1). The gut microbiota encompasses both gram-positive and gram-negative bacteria, the latter expressing a structural component called lipopolysaccharide (LPS) that because of its ability of acting as a toxin is also described as an endotoxin (4). Because of its strong ability to activate an immune response, endotoxin is one of the possible links between gut bacteria and immune cells in the gut–brain axis. The level of endotoxin can be quantified in a qualitative fashion with a gel-clot assay, or quantitatively using chromogenic assays or kinetics assays. The latter are the most sensitive and reliable because they are less affected by technical artifacts (5).

The quantification of the levels of endotoxin is common in the pharmaceutical field to validate the purity of drugs and medical devices. The measurement of endotoxin in biological fluids as serum is hindered by esterases, fibrinogen, and high-density lipoproteins that can inactivate the endotoxin thus preventing the activation of the enzyme used in the assay—this was discussed at greater extent from Hurley and colleagues (5). They interestingly described how heat inactivation, the use of a kinetic assay, and proper sample dilution can help to avoid the inhibitory activity and background color faced with blood samples (5).

Two studies investigated the presence of endotoxin in the blood of multiple sclerosis (MS) patients and showed that plasma endotoxin is increased in MS patients compared with healthy controls (HC) (6, 7); of note there was a visible overlap between the two groups, and Teixeira and colleagues also reported an association between the endotoxin levels and the level of disability of the patients measured by the Expanded Disability Status Scale (EDSS) (6). Nonetheless, these studies used a non-kinetic chromogenic assay that was not recommended nor validated for the quantification of endotoxin in blood. The quantification of endotoxin in blood can be attempted by more reliable kinetic assays. We here investigated and compared the analytical performance of two kinetic assays: the Limulus Amebocyte Lysate (LAL) Kinetic-QCL (#50-650U; Lonza, MD, USA) and the turbidimetric LAL Pyrogent-5000 (#N384; Lonza). In the QCL assay, the endotoxin present in the sample activates a proenzyme to produce *p*-nitroaniline which rate of increase is proportional to the level of endotoxin and can be measured at 405 nm (Manual LAL Kinetic-QCL RT-MN008). The Pyrogent-5000 assay also takes advantage of the level of endotoxin for activating an enzyme to produce coagulin that forms clots. The clot formation is visualized as turbidity and its optical density is measured at 340 nm wavelength. The technical advantage of these kinetic assays over the classical one-time read is that they look at the increase in signal over time, hence the final readout is not affected by the initial color of the sample and it can easily be appreciated if the sample is in the linear range of the assay. Moreover, the presence of EDTA, citrate, and heparin that are used for collecting plasma samples can also affect the detection of endotoxin (8). Because of this, we decided to use serum samples. Here, we performed an analytical validation of two kinetic assays and investigated the levels of endotoxin in serum samples of patients with multiple sclerosis (MS) and healthy controls.

## Methods

### Sample Population

The initial validation was performed on serum samples from two healthy donors. The analysis of clinical samples included samples from 8 HCs, 10 RRMS, and 10 SPMS patients as defined by the 2010 McDonald criteria (9). The samples were from our CLIMB biorepository; samples were collected in red top serum Vacutainer tubes (glass, silicone coated, no additives), left at room temperature for 30–60 min to allow the clot to form. The tubes were then centrifuged at 4°C, 2,000 rpm for 10 min and serum was collected under sterile conditions and frozen at −80°C until measurement. The patients were treatment naïve and the three groups were age matched. Informed consent and EDSS scores were obtained from patients in an ongoing observational cohort study of MS patients at the Partners MS Center (CLIMB study). Institutional Review Board approval was granted by the Partners Human Research Committee, and participants provided written informed consent for participation.

### Endotoxin Measurements

We quantified endotoxin in serum samples by using the Lonza Kinetic-QCL (#50-650U; Lonza) and Lonza Pyrogent-5000 (#N384; Lonza) assays. To avoid contamination, all procedure used pyrogen-free materials including pyrogen-free tips (Eppendorf Biopur tips 200 μl, #25-115, Lonza and 1,000 μl, #25-417, Lonza). The assays were run on a Biotek Epoch 2 reader using the Software Gen5 2.09. The procedure followed the instructions provided by the vendor. Briefly, samples were diluted 1:10 in endotoxin-free water (EFW, LAL Reagent Water 100 ml, #W50-100; Lonza) using pyrogen-free microcentrifuge tubes (United Scientific Supplies Micro Centrifuge Tubes, #S13082; Fisher Scientific) and heat treated at 70°C on a heat block. Calibrators were prepared by serial dilution of *E. coli* O55:B5 (Lonza, #7460) with EFW in borosilicate glass tubes (pyrogen-free dilution 12 × 100 mm without caps, #N207; Lonza). The blank was 100 μl of EFW as per vendor instructions. A volume of 100 μl of sample and calibrators were plated in duplicate on a pyrogen-free 96-well plate (LAL reagent grade multi-well plated, #25-340; Lonza). The plate was placed in the BioTek reader and incubated at 37°C for 10 min. During this incubation time, the needed number of Kinetic-QCL or Pyrogent-5000 Reagent vials was reconstituted in EFW or Pyrogent-5000 Reconstitution Buffer, respectively. At the end of the incubation, using a multichannel pipette and pyrogen-free reagent reservoir (LAL Reagent Reservoirs, #00190035; Lonza), 100 μl of reagent was added in each well and the reading was started. The Kinetic-QCL assay was read at 405 nm every 2 min and 30 s and the Pyrogent-5000 at 340 nm every 60 s. Both assays were run for 2 h in a constant temperature of 37°C. The increase in signal of calibrators and samples was plotted against time; both Delta signal and concentration were log transformed. The slopes of the calibrator curves were modeled with a 4PL equation and the concentrations of the samples were interpolated on this curve using the Gen5 2.09 software. The acceptance criteria were defined as a spike recovery between 50 and 200% as defined by the United States Pharmacopeia and commonly used in the field (10) and a recovery for the serial dilution and parallelism between 70 and 130% that is the threshold commonly used in immune-assay validation (11).

### Statistical Analysis

We compared the endotoxin levels between HC, RRMS, and SPMS by Mann–Whitney *U* test. We investigated the correlation between the endotoxin levels and EDSS or age by non-parametric Spearman correlation.

## Results

### Heat Treatment

**Figure 1:**
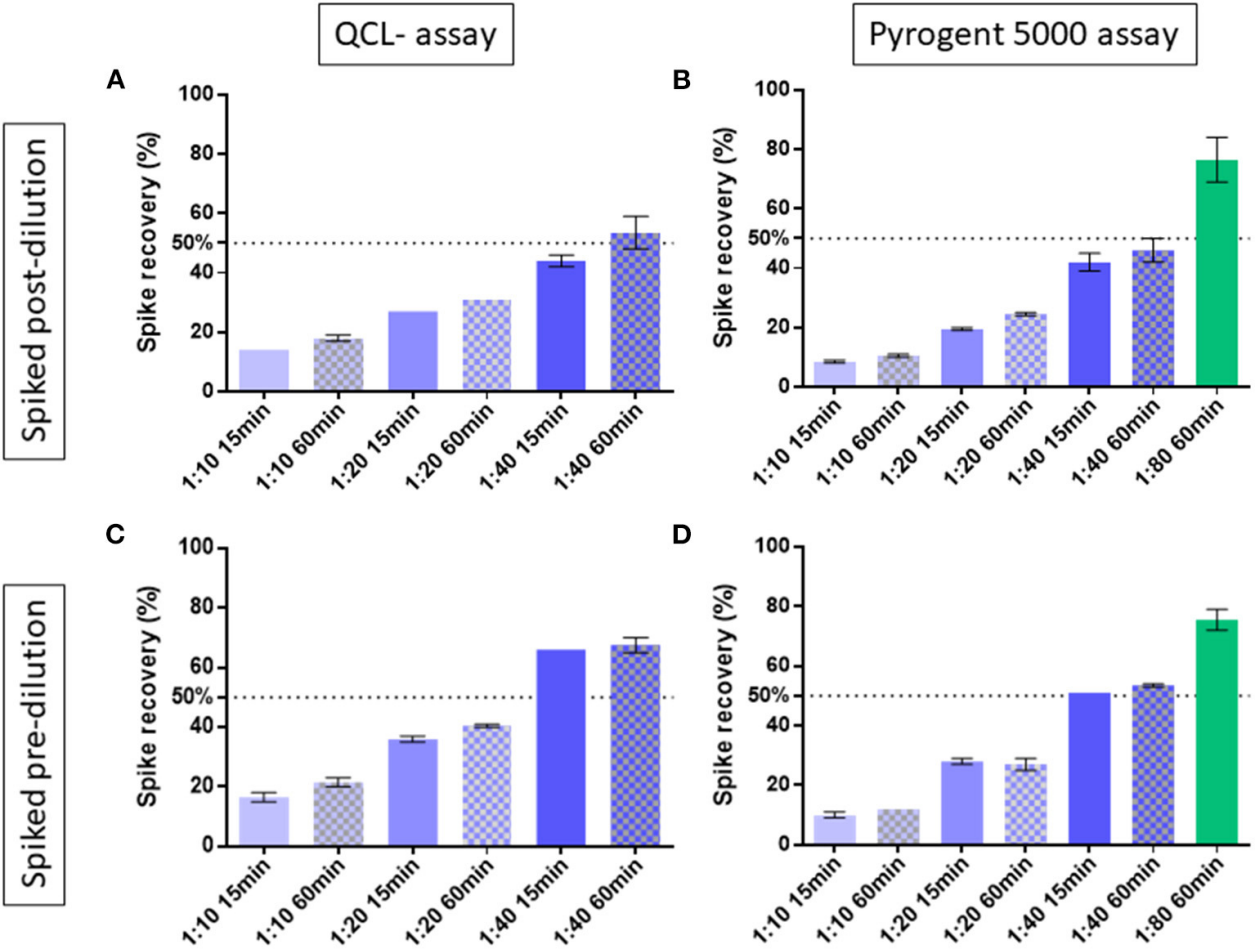
Spike recovery in two human serum samples exposed at 15 or 60 min of heat treatment and measured at different dilutions. The best condition of 1:80 dilution with 60 min of heat treatment was tested with the Pyrogent-5000 assay. Median and interquartile range are shown. A line at 50% shows the limit of acceptance as defined by the United Stated Pharmacopeia (10). Samples were spiked with endotoxin after their final dilution and measured using **(A)** the QCL assay and **(B)** the Pyrogent-5000 assay. Samples were spiked with endotoxin before dilution and before heat treatment and measured **(C)** with the QCL assay and **(D)** with the Pyrogent-5000 assay.

We exposed the samples to 70°C on a heat block for 15 or 60 min. As suggested by the vendor (Lonza assay tips RT-DS006), the samples were diluted 1:10 in EFW before heat treatment. The spike recovery post-dilution, which is the gold standard measure for validating endotoxin measurements (10), showed consistently better performance with the 60-min compared with the 15-min heat treatment throughout the different dilutions. For instance, the QCL assay with 1:40 dilution resulted in an average spike recovery of 44.0% with 15-min and 53.5% with 60-min heat treatment. At the same 1:40 dilution, the Pyrogent-5000 showed a 42.0 and 46.0% spike recovery with respectively 15 and 60 min of heat treatment (Figures 1A,B).

### Dilution Linearity

We first investigated assay linearity (12) by measuring the samples at 1:10, 1:20, and 1:40 dilution with both the QCL and Pyrogent-5000 assay in two spiked samples. The percent recovery was calculated for the 1:20 dilution on the levels of the 1:10 dilution, and for the 1:40 dilution on the levels of the 1:20 dilution after back calculating for their dilution, for instance:

(1) $$\begin{array}{r} Recovery\;\left(\%\right):\;\frac{\left(Endotoxin\;at\;1:20\right)\;\times \;20}{\left(Endotoxin\;at\;1:10\right)\times \;10}\times 100 \end{array}$$

**Figure 2:**
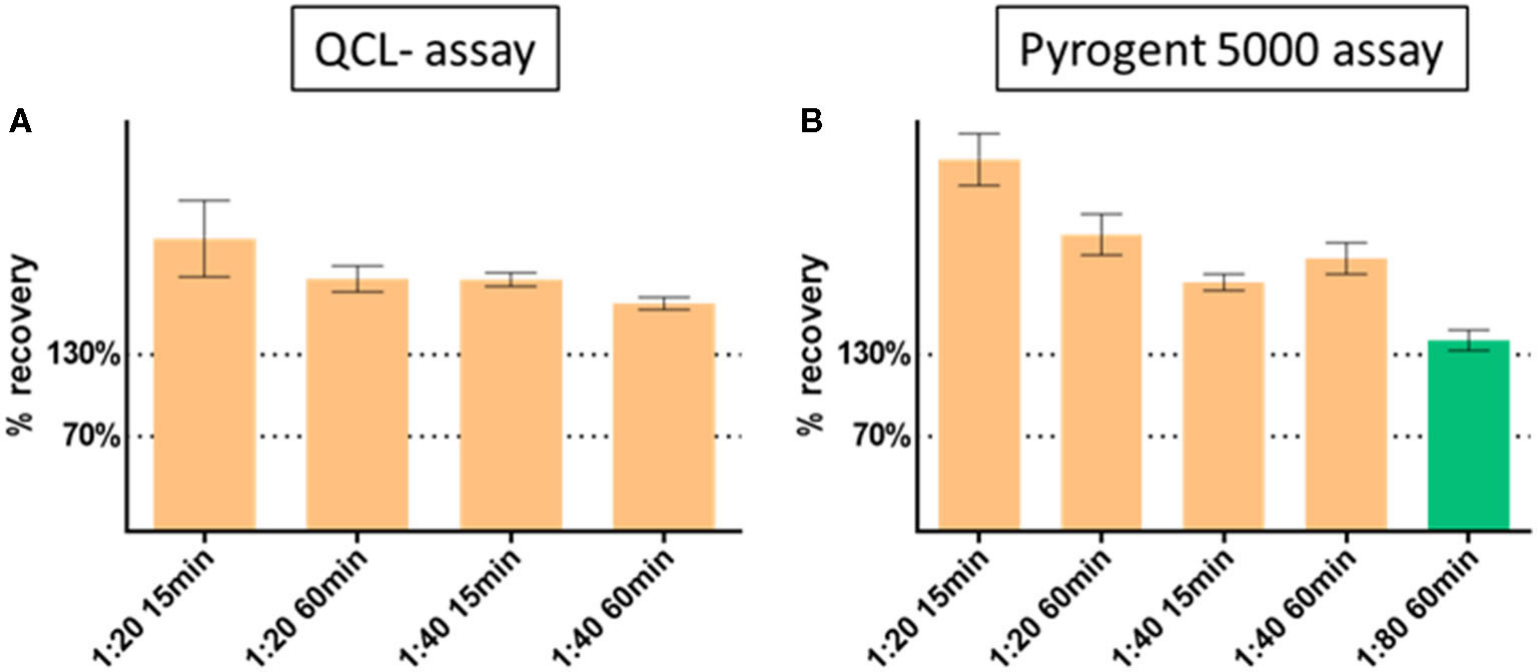
Dilution linearity with **(A)** QCL and **(B)** Pyrogent 5000 kinetic assays. Spiked samples were serially diluted at 1:10 (reference at 100%), 1:20, 1:40 with endotoxin free water. The dilution 1:80 was further explored on the Pyrogent 5000 assay. Samples were heat treated on a heat block at 70°C for 15 or 60 min.

The dilution linearity showed increasing performance with increasing dilution (Figure 2). Because of the Pyrogent-5000's better performance, we explored a further 1:80 dilution with this assay. The latter dilution showed an improved recovery performance by reaching the upper limit of the expected range of 70–130% and hence could be defined as candidate minimal required dilution for the assay (Figure 2). We validated this further in the parallelism section.

### Spike Recovery

To assess a possible difference between the sample matrix and the calibrator matrix and a possible residual inhibitory activity in the sample, we spiked the samples pre- and post-dilution with *E. coli* O55:B5 Endotoxin (Lonza, #7460). Considering the detection range of the assays, we spiked 1 EU/ml pre- or post-dilution for the QCL assay and 10 EU/ml pre-dilution or 0.4 EU/ml post-dilution for the Pyrogent-5000 assay. The percent spike recovery was calculated by subtracting the concentration of the unspiked sample from the spiked samples and dividing this by the amount that was spiked:

(2) $$\begin{array}{r} Spike\;recovery\;\left(\%\right):\\ \frac{\left(Endotoxin\;spiked\;sample-Endotoxin\;unspiked\;sample\right)}{Spiked\;amount}\times 100 \end{array}$$

The spiking post-dilution fulfilled the acceptance criteria with a 1:40 dilution for the QCL assay (53.5%) and with a 1:80 dilution for the Pyrogent-5000 assay (76.5%) (Figures 1A,B). On the other hand, the spiking pre-dilution, which is more sensitive to physical properties of the serum matrix, achieved the acceptance range with a 1:40 dilution for both assays (67.5% for the QCL and 53.5% for the Pyrogent-5000) (Figures 1C,D).

### Calibrators

*Lower limit of detection* (LLOD) was defined as the lowest calibrator having a %CV below 20%. The LLOD was 0.005 EU/ml for both assays. *Lower limit of quantification* (LLOQ) was 0.005 EU/ml multiplied for the dilution factor of the samples (1:80) that is 0.4 EU/ml for both assays.

**Figure 3:**
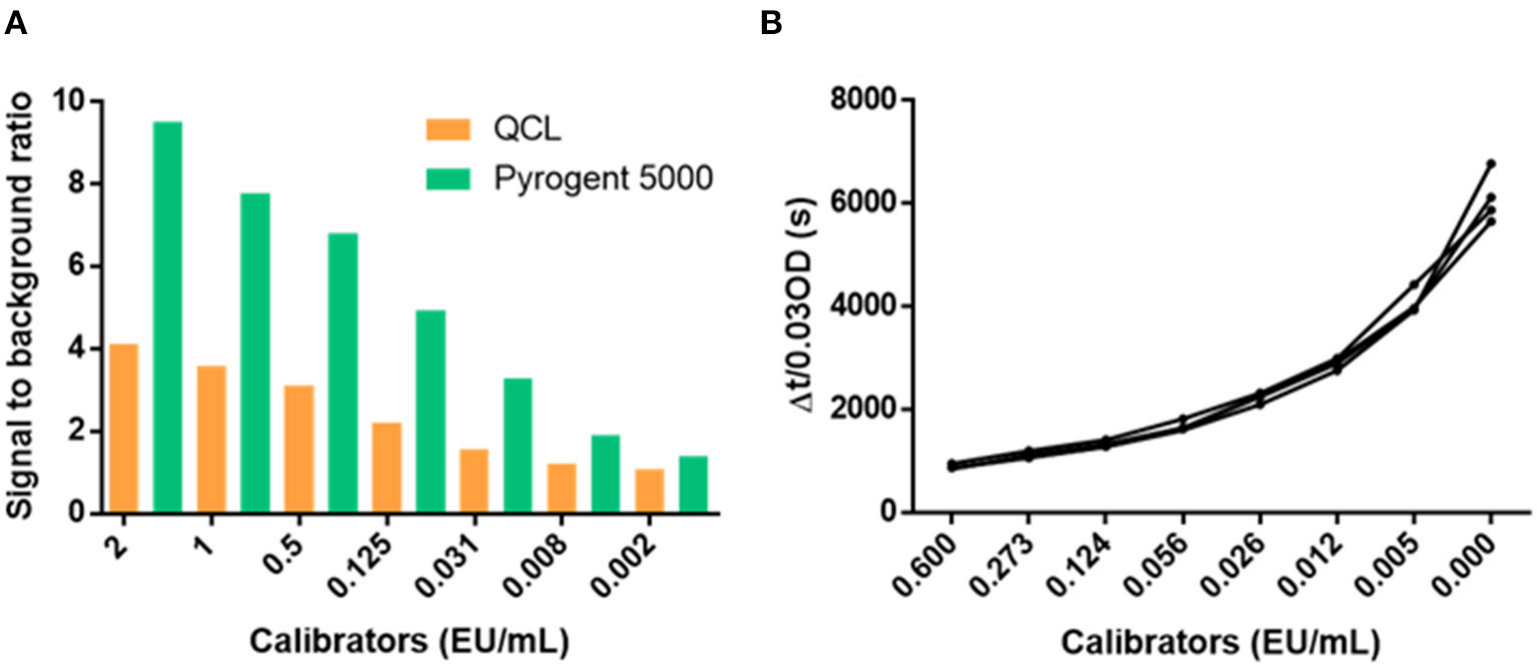
Performance of the calibrator curves. **(A)** Signal to background ratio of the calibrators. **(B)** Reproducibility of the calibrators over four runs performed in different days. CV, coefficient of variation.

We performed experimental runs with the same levels of calibrators in both the QCL and Pyrogent-5000 assay to compare their performance. The Pyrogent-5000 assay showed a better signal to noise with an average of 4.58 compared with the 2.24 of the QCL assay (Figure 3A). Considering the similar performance obtained in the dilution linearity and spiking experiments, we deemed the Pyrogent-5000 assay as more suitable for measuring clinical samples. Moreover, the calibrators were repeatedly measured on four different days and obtained an excellent reproducibility with only an average 5% CV (Figure 3B).

### Parallelism

**Figure 4:**
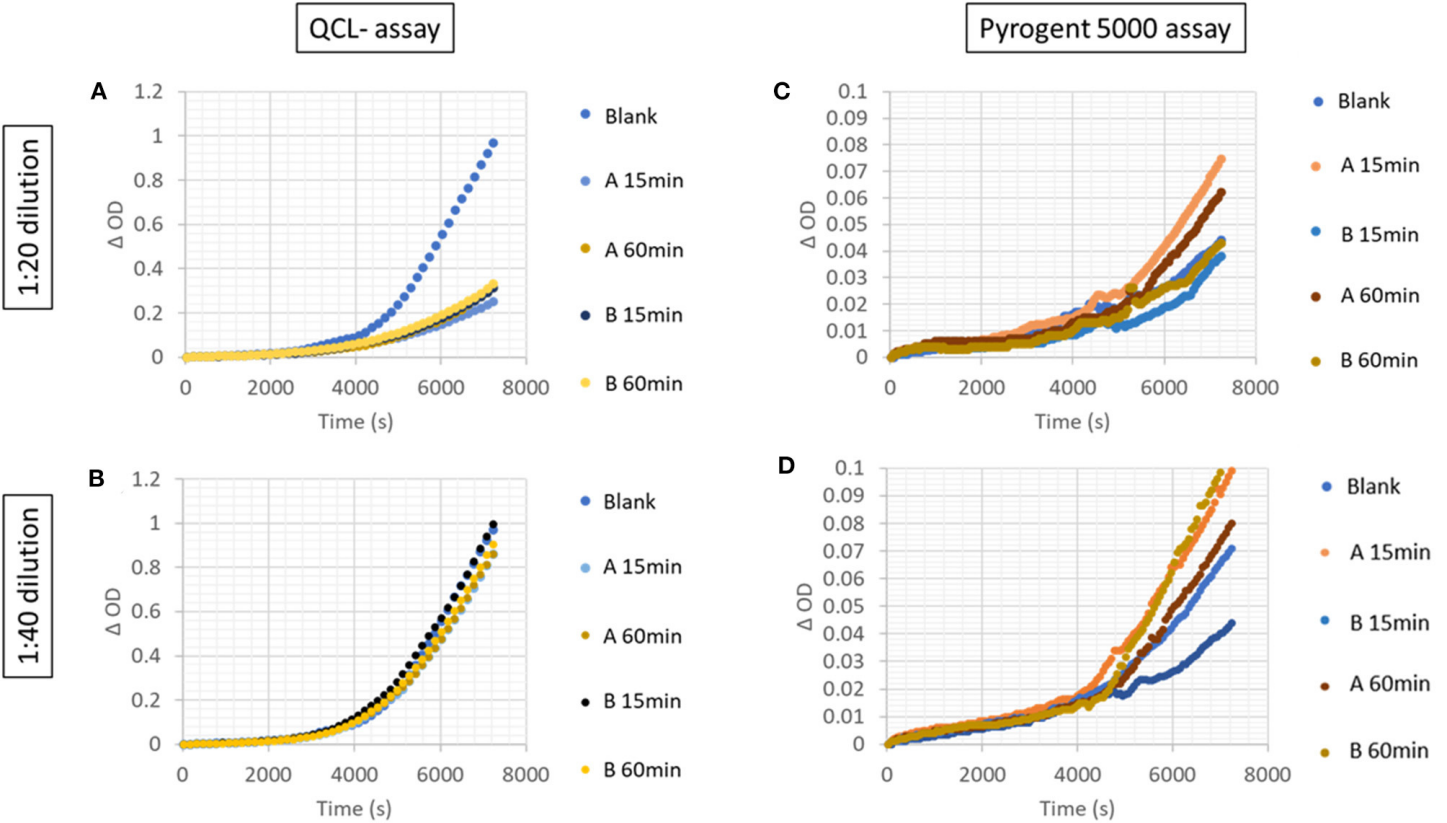
Increase in signal over time in serially diluted unspiked serum samples with the **(A,B)** QCL and **(C,D)** Pyrogent-5000 assays.

**Figure 5:**
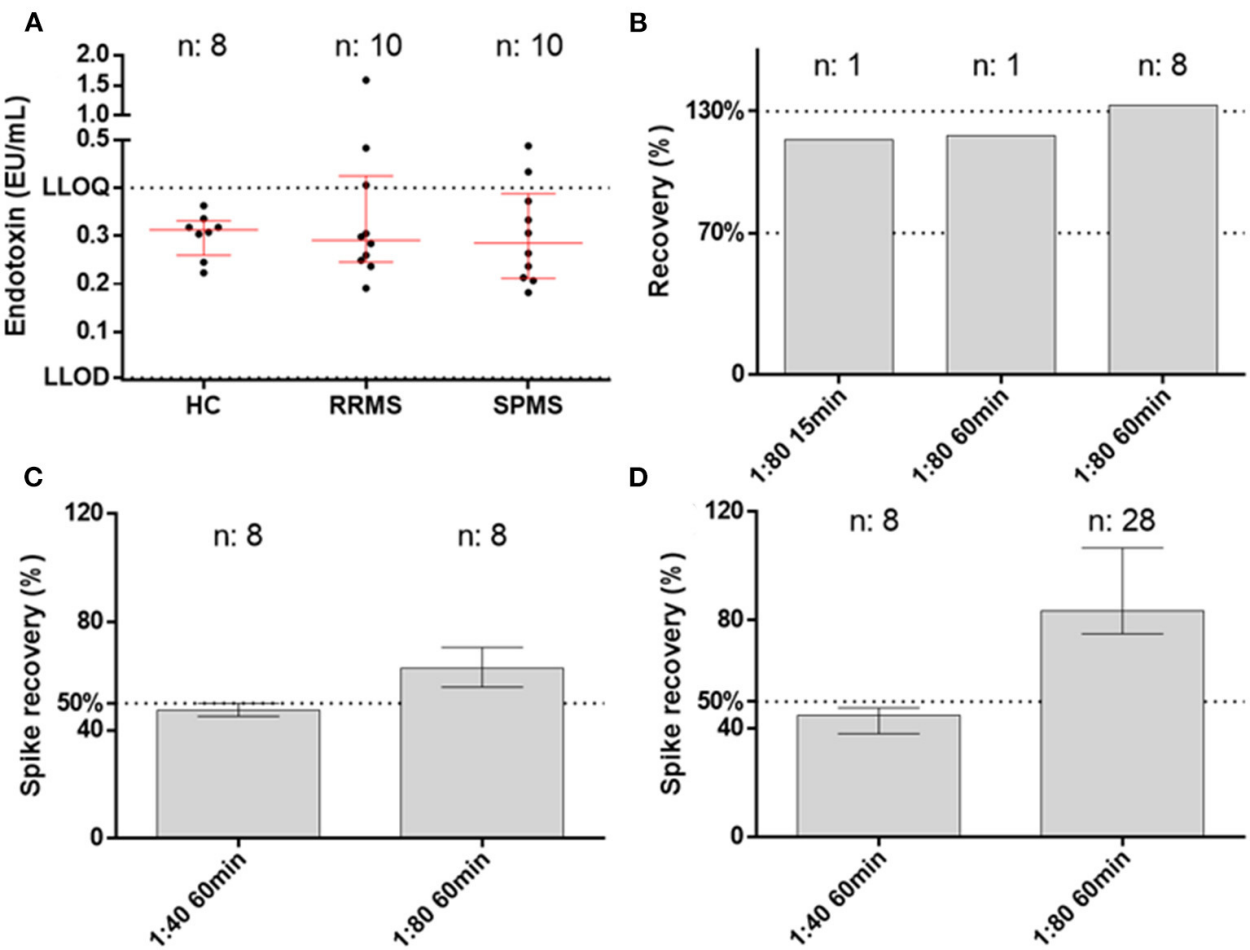
Endotoxin levels in samples and assay performance. **(A)** Endotoxin levels in healthy controls (HC), relapsing–remitting multiple sclerosis patients (RRMS), and secondary progressive multiple sclerosis patients (SPMS) measured at the best conditions of 1:80 dilution and 60 min of heat treatment. **(B)** Parallelism for the native sample with both 15 and 60 min of heat treatment. The third column shows the dilution linearity on spiked samples that remained at the upper level of the accepted range (70–130%). **(C,D)** Spike recovery pre- and post-dilution.

Two native (not-spiked) serum samples measured with a 1:20 dilution on the QCL assay showed a lower increase in signal than the blank (Figure 4A). Hence, the samples had a residual inhibitory activity that hampered the assay reaction, which was overcome by a higher dilution, e.g., 1:40 dilution (Figure 4B). This inhibitory activity was not observed on the Pyrogent-5000 for the same dilutions (Figures 4C,D). Moreover, we identified one serum sample with a native endotoxin concentration above the LLOQ of 0.4 EU/ml. We diluted this native serum sample 1:40 or 1:80 and percent recovery was calculated after adjusting for the dilution factor with the same equation described in the serial dilution section. This was done with either 15 or 60 min of heat treatment. The minimal required dilution was defined as the lowest dilution needed for complying with the acceptance criteria of a recovery between 70 and 130%. The 1:80 dilution, which we identified with the dilution linearity experiments, confirmed to be ideal and achieved a recovery of 116 and 118% for 15 and 60 min of heat treatment, respectively (Figure 5B).

### Analyte Stability

A serum sample with an elevated native endotoxin concentration was aliquoted and froze in −80°C for 4 months and after this time it was remeasured. The concentration obtained was in the first run 1.588 EU/ml and in the second run 1.491 EU/ml, corresponding to an excellent inter-assay %CV of 4.4%. Thus, the analyte was stable at −80°C and the assay showed an excellent stability over time.

### Clinical Samples

We took advantage of the best conditions obtained during the validation (1:80 dilution and 60 min of heat treatment with Pyrogent-5000 assay) for measuring clinical samples from healthy volunteers and MS patients (Table 1). The levels of endotoxin did not differ between the groups with a median level of 0.3128 EU/ml in HCs, 0.2910 EU/ml in RRMS, and 0.2850 EU/ml in SPMS patients (Figure 5A). There were zero samples from healthy controls, three from RRMS, and two from SPMS patients above the LLOQ (0.4 EU/ml). Overall, 5 out of 20 samples from MS patients had levels above the LLOQ compared with zero samples from the age-matched healthy control group. There was no association with age or EDSS. The measurements were valid as defined by both spiking pre- and post-dilution within the accepted range (50–200%) (10), hence the validated conditions confirmed to be suitable for performing the measurement of endotoxin in serum (Figures 5B–D).

**Table 1:** Patients and healthy controls included in the study.

**Variables**	**HC**	**RRMS**	**SPMS**
*N*	8	10	10
Age (years)	57.5 (44.3–71.8)	57.5 (48.8–66.2)	56.7 (41.3–65.5)
Sex (F %)	38%	50%	80%
EDSS	–	1.9 (1.0–3.0)	5.4 (2.0–9.0)
Treatment	–	Treatment naïve	Treatment naïve
Endotoxin (EU/ml)	0.31 (0.26–0.33)	0.29 (0.25–0.43)	0.29 (0.21–0.39)

*Mean and range for age and EDSS. Median and interquartile range for endotoxin levels. EDSS, expanded disability status scale*.

## Discussion

We optimized and validated two kinetic assays for performing the measurement of endotoxin in serum samples. A measurement of endotoxin is usually referred as valid if it reaches a spike recovery above 50% because the reaction was not interfered by the activity of inhibitory enzymes (10). Interestingly, in the field of immune assay, it is more common to spike the native samples (pre-dilution) to verify the presence of a matrix effect—in this case serum matrix—that can hinder the detection of the analyte. Both assays performed well in spiking pre- and post-dilution with at 1:40/1:80 dilution and a prolonged heat treatment of 60 min appeared to be beneficial to the assay performance compared with a shorter heat treatment of 15 min. Moreover, the Pyrogent-5000 showed a better signal to noise and hence a better sensitivity to detect endotoxin levels compared with the QCL assay. Thus, we took advantage of the best-performing conditions and assay, the Pyrogent-5000, and we investigated whether the levels of endotoxin are increased in treatment-naïve relapsing or progressive MS patients compared with healthy controls. We did not find a significant difference between the three groups; hence, we did not find evidence to support a penetration of endotoxin in the blood of MS patients. Nevertheless, the lack of endotoxin in the serum does not exclude its potential role at other anatomical locations such as at the gut–blood barrier where the communication between bacteria and immune system happens locally through antigen-presenting cells [such as dendritic cells (13)] and therefore does not require metabolites or bacterial components such as endotoxin to enter the blood circulation. Of note, 25% (5 out of 20) of the MS patients had levels above the LLOQ (0.4 EU/ml) that compares with 0% of the healthy control samples. Nonetheless, only one sample from a RRMS patient had an appreciable increased level of endotoxin with 1.5 EU/ml. The levels that we found in the serum of healthy controls and in the majority of patients are in line with the levels described in literature for healthy individuals of 0.1 ± 0.2 EU/ml (4). There are two previous studies that explored the levels of endotoxin in the plasma of MS patients and found that the levels were increased in MS patients compared with controls (6, 7) and were associated with EDSS (6). We did find that more samples from MS patients are above the LLOQ, but the difference between the groups was not significant. Moreover, we did not confirm the correlation with the EDSS. There is an increasing interest in measurement of blood endotoxin levels also in patients with HIV because they are known to have a higher permeability at the gut barrier caused by the depletion of the T-cell compartment. Few studies explored the plasma and cerebrospinal fluid (CSF) levels of endotoxin in HIV patients by using an endpoint chromogenic LAL assay (Lonza, the category number was not specified but based on the vendor it is most probably the QCL-1000 assay). The endotoxin levels were below the detection limit in the CSF but were detectable and increased in plasma samples of the untreated group (14, 15) or the patients who developed hypertension (16). The mentioned studies in both MS (6, 7) and HIV (14, 15) used (1) a non-kinetic chromogenic assay that as discussed is prone to interference (17) and is affected by the baseline color of the samples and (2) used plasma samples—plasma was shown to influence the readout of the endotoxin level (8). We performed our measurements leveraging reliable and sensitive kinetic assays and used serum instead of plasma samples. The advantages of a kinetic assay over an endpoint chromogenic assay is (1) the possibility to monitor the reaction over time, thus we can identify the linear range of the measurements by kinetic plots with the change in OD over time and (2) the ability to avoid the bias given by the initial absorbance defined by intensity of the color of the sample. We addressed the possibility that the serum matrix may have hampered the detection of endotoxin by performing several tests of the spike recovery in both undiluted and diluted samples showing that the serum matrix is suitable for performing the assay. Hence, the discrepancy between our results and others that looked at plasma of MS patients is probably caused by a difference in the method used to quantify endotoxin or hypothetically also the different collection method between serum and plasma may have originated this discrepancy.

## Conclusion

Our study supports the utility of kinetic assays for a reliable quantitative detection of endotoxin in serum samples. We showed that a minimal required dilution of 1:80 is necessary for obtaining optimal analytical performances and that these performances are improved by a prolonged heat treatment duration of 60 min. We did not find increased endotoxin levels in the serum of MS patients compared with controls. Of note, we cannot exclude the possibility that the collection of the serum fraction of the blood is inherently affecting the levels of endotoxin. From the data we obtained from serum samples, endotoxin does not seem to enter the blood circulation in MS patients. Its possible involvement in the crosstalk between the gut microbiota and the immune system in multiple sclerosis patients might be limited locally at the level of the gut–blood barrier.

## Data Availability Statement

The raw data supporting the conclusions of this article will be made available by the authors, without undue reservation.

## Ethics Statement

The studies involving human participants were reviewed and approved by Partners Human Research Committee. The patients/participants provided their written informed consent to participate in this study.

## Author Contributions

CB: study design, literature search, data generation, data analysis, data interpretation, and writing. AP, FS, TC, and HW: sample collection, literature search, data interpretation, and critical review of article. All authors contributed to the article and approved the submitted version.

## Conflict of Interest

TC has received compensation for consulting from Biogen, Novartis Pharmaceuticals, Roche Genentech, and Sanofi Genzyme. She has received research support from the National Institutes of Health, National MS Society, US Department of Defense, EMD Serono, I-Mab Biopharma, Mallinckrodt ARD, Novartis Pharmaceuticals, Octave Bioscience, Roche Genentech, and Tiziana Life Sciences. HW reports grants from National Institutes of Health, National Multiple Sclerosis Society, Verily Life Sciences, Google Life Sciences, EMD Serono, Inc., Biogen, Teva Pharmaceuticals, and Novartis; grants and consulting fees from Sanofi US Services, Inc. and Genentech, Inc.; consulting and advising fees from Tilos Therapeutics; consulting and advising fees from Tiziana Life Sciences; consulting and advising fees from IM Therapeutics; personal, consulting and advising fees from vTv Therapeutics; personal, consulting and advising fees from MedDay Pharmaceuticals. The remaining authors declare that the research was conducted in the absence of any commercial or financial relationships that could be construed as a potential conflict of interest.
